# Microparticulate Cortical Allograft: An Alternative to Autograft in the Treatment of Osseous Defects

**DOI:** 10.2174/1874325000802010091

**Published:** 2008-05-14

**Authors:** H. Thomas Temple, Theodore I Malinin

**Affiliations:** Tissue Bank, Department of Orthopaedics, University of Miami, Miller School of Medicine, Miami, Florida, USA

## Abstract

Benign bone tumors are commonly diagnosed and treated. Following tumor removal, the defect in the bone can be filled with auto- or allografts, (degradable) bone substitutes or non-degradable polymethylmethacrylate. The ideal substitute for this purpose should provide immediate structural support and readily incorporate into bone over a short period of time. Experimentally, microparticulate allograft has been shown to incorporate quickly in metaphyseal and metadiaphyseal cortico-cancellous defects in primates [1]. Using a combination of small allogeneic cortical graft particles (< 250 µm), bone defects were filled following intralesional excision in 97 consecutive patients with benign and low grade malignant tumors and tumor-like conditions of bone. The clinical results and rate of radiographic incorporation and osseous consolidation were recorded and analyzed. These patients underwent 104 procedures in which osseous defects were packed with microparticulate allograft. The follow-up was from 23 to 49 months. There were 94 (90.3%) closed defects and 10 (9.7%) open defects. The average size of the grafted defect was 42.8 cm^3^ (0.48 - 315.0 cm^3^). Internal fixation was used in 11 of the 104 procedures (10.6 %). Radiographically, incorporation was observed in 91% of patients and consolidation in 60%. There were eleven failures (10.6 %), eight (72 %) due to tumor recurrence. Seven of eight patients with tumor recurrence underwent a second resection and grafting procedure that resulted in allograft incorporation and defect healing. There were two deep infections requiring debridement with retention of the graft; both resolved with satisfactory healing.

Both incorporation and consolidation were observed in over 90% of patients with a low rate of complications. The use of small-particle cortical allograft proved to be an effective alternative to autogenous bone graft in patients with metaphyseal and metadiaphyseal surgical bone defects.

## INTRODUCTION

Benign bone tumors and tumor-like conditions of bone are rather common. After these lesions are surgically removed the defects are usually filled with either an autograft, an allograft, a variety of bone substitutes or polymethylmethacrylate. Ideally, the material selected should be both osteoconductive and osteoinductive, it should provide sufficient early mechanical stability, and it should allow the regeneration of normal bone in a short period of time.

Advances in surgical technology, during the latter half of the twentieth century, have coincided with an increase in the use of new materials and complex surgical procedures for bone repair [2-6]. Autogenous bone is still considered to be the gold standard for bone grafting. It provides three elements necessary to generate and maintain bone: scaffolding for osteoconduction, growth factors for osteoinduction, and progenitor cells for osteogenesis. Cancellous autograft, however, is in limited supply and its harvesting can result in significant donor site morbidity. Allografts, on the other hand, may not have similar osteoinductive properties to autografts, depending on the way these grafts have been processed. Since the means of preparation and storage, and the structural form of the allograft may significantly affect graft incorporation, its use has been associated with mixed clinical results. Therefore in the past several years, there has been an explosion in the development of bone substitutes, growth factors, and combinations of the two that have made their way to the medical market [3,4]. Well designed clinical trials, however, have not yet demonstrated their efficacy or safety [2].

As the orthopaedic oncologist’s ability to achieve local control of bone tumors improves, emphasis is placed on healing of the bone cavity to achieve early function [5, 7]. For most benign tumors, intralesional curetting and bone grafting is the treatment of choice [8]. The materials used to achieve this are: autograft, bone substitutes, cement, and allograft.

The usefulness of autograft is limited by availability and donor morbidity. Donor site complications, infection and lateral femoral cutaneous nerve injury, as well as structural insufficiency that can lead to fracture may result in increased patient recovery time, disability, and chronic pain [3]. Furthermore, the residual pain and disability that may result from harvesting cancellous iliac crest bone can be relatively long-lasting. These problems justify continued efforts to find effective bone graft substitutes as alternatives to autografts [4, 9].

Biocompatible substitute materials, such as porous hydroxyapatite, tricalcium phosphate, etc offer the surgeon additional choices in the repair of cavitary long bone defects [3, 6]. The ideal composite synthetic grafts should effectively combine the three salient bone-forming properties (osteoinduction, osteoconduction and progenitor cells) [6]. The drawbacks of many substitutes include low biocompatibility, poor restorability, inclusion of processed animal components, inferior handling characteristics, and cost. In clinical practice, incomplete healing has occurred commonly in patients with large lesions [5]. Further investigation, in animal models, evaluating different defect sizes and biomechanical conditions will help to determine the appropriate use for these materials [10, 11].

Polymethymethacrylate (PMMA) has been used to fill large subchondral defects following removal of giant cell tumors.. Once polymerized, PMMA resists axial loading and thus provides immediate structural support. Moreover, the exothermic reaction during polymerization is thought by some to extend the margin of resection. PMMA, however, has no biologic activity and cannot remodel in the face of changing stresses. Therefore, it is less desirable for the treatment of curable lesions when the goal is complete restoration of anatomy and function [12].

Allograft bone has now been used clinically for more than 50 years for various orthopaedic indications [13-16]. Allografts are available in reasonable quantities and are versatile since shape, contour, and mineral density can be altered and tailored to a particular clinical indication. Allograft structure is very important when considering the type of defect and the means by which the graft can be expected to incorporate over time. Materials such as DBM (Demineralized Bone Matrix), DFDBA (Demineralized Freeze-Dried Bone Allograft), allograft paste, powder, chips, strips and putty, all have different mechanical and osteoinductive properties [17-20]. Osteoinductive properties of allograft bone can be altered by many different secondary sterilization techniques. Allograft chips, when used to fill acetabular defects in total hip revision surgery, have shown, in microscopic preparations, to incorporate within 3 weeks [14, 19]. Massive allografts, incompletely incorporate from the periphery inward by a process of creeping substitution [21]. Unpredictable clinical results have been reported with these for a variety of reasons [15, 18]. On the other hand cortical microparticulate allograft incorporates rapidly due to exposed inductive proteins from the large trabecular surface area and interconnected spaces [1, 9]. Furthermore, due to the small particle size, the resorptive phase of bone healing is accelerated.

Independent of the type of graft used, the size of graft particles has an important effect on incorporation [1, 22, 23]. Thus, Shapoff, *et al. *[22] demonstrated increased osteogenesis with small particle, freeze-dried bone allograft when compared to larger particles. However the studies of Syftestad an Urist [23], showed that very small particle sizes (under 125 μm) provided an increase in surface area that enhanced the generation of mechanically-induced free radicals resulting in increased solubility of bone matrix proteins and decreased bone production.

Allograft particle size is also related to resistance to shear stresses. *In vitro* experiments, using morsellised allograft bone, have shown that the graft’s mechanical properties improve with increasing normal load and with increasing shear strains (strain hardening) [24]. Further studies were needed to determine the optimal allograft particle size for filling bone defects. These were provided by the experiments on non-human primates and were applied to the present clinical investigation [1, 7].

Optimal osteoconduction is provided by direct apposition between host bone and the implant. The host bone must be viable, the host bone/implant interface must be stable and the implant needs to have a structure (porosity) that allows new bone in-growth. Also important is the fact that optimal incorporation of the graft occurs when histocompatibility differences are minimized by either matching tissue types or by treating the allograft with techniques that reduce immunogenicity [13]. Although freezing significantly reduces antigenicity and thus the inflammatory response it does not reduce it as much as does freeze-drying [7]. Furthermore, although allografts pose a known risk of bacterial contamination and viral transmission, rigorous tissue screening and microbiologic and serologic testing of donor tissue reduce these risks to theoretical possibilities [13].

The microparticulate allograft is a mixture of mineralized cortical bone particles of a standard combination of sizes between 100 and than 250 microns. This provides an optimal scaffold for delivering osteoinductive growth factors. This preparation has an appropriate 3-dimensional (3-D) structure to serve as an osteoconductive matrix for bone-forming cells.

The purpose of this study was to evaluate, in a prospective, consecutive series of patients, the results obtained with freeze-dried small-particle cortical allograft as an alternative for autogenous bone graft in filling bone defects created by tumor ablation. Our hypothesis was that freeze-dried cortical microparticulate allografts would induce healing of bone defects in a manner similar to autografts. Freeze-dried microparticulate allografts were chosen because they were shown to incorporate faster than frozen microparticulate allografts [7]. Likewise cortical bone particulate allografts with particle sizes from 300 to 90 µ were shown experimentally to produce healing of bone defects in a manner and rate that was identical to that produced by autografts [1].

## MATERIALS AND METHODS

Ninety seven consecutive patients with benign and low grade malignant bone tumors and tumor-like conditions of bone, aged 12 to 75 years (35.2 years mean), underwent a total of 104 procedures between January 2000 and October 2003. There were 53 females (50%) and 44 males (46%) Patients had pre-operative imaging studies that included radiographs, computerized tomography, magnetic resonance imaging and bone scintigraphy when appropriate. Needle biopsies were performed pre-operatively in some patients, but most patients underwent an open biopsy at the time of definitive surgery to confirm the diagnosis. Tumors were classified as benign latent, benign aggressive, and low-grade malignant.

**Fig. (1):**
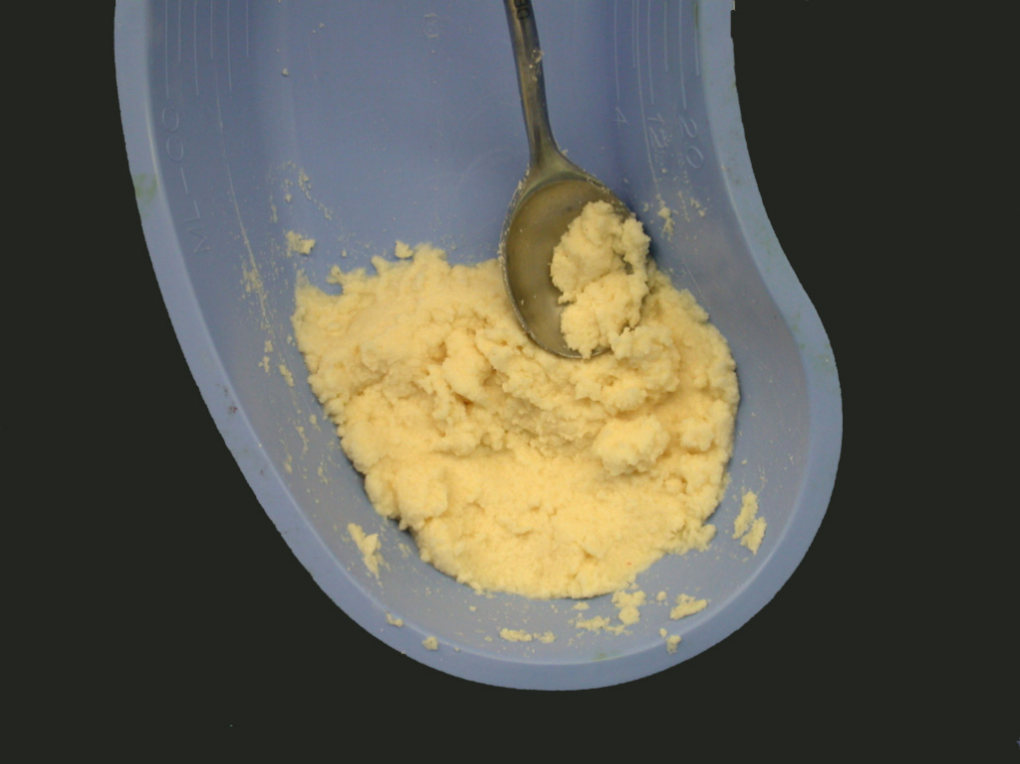
Cortical microparticulate allograft reconstituted with saline.

The microparticulate cortical bone allograft was prepared by the University of Miami Tissue Bank (Miami, FL) according to a previously described method (7). This allograft was generated from a single donor. Freeze-dried cortical bone particles were less than 250 μm in maximum dimension and over 100 mm in minimum dimension. All bones from which allografts were prepared were excised and processed aseptically. The allografts were processed in compliance with Food and Drug Administration and American Association of Tissue Banks standards. The microparticulate allograft was reconstituted with sterile saline (Fig. **1**) or left in its original state, and packed or injected into the defect under fluoroscopy. The graft was injected percutaneously or packed through an open wound.

Biplanar radiographs of the operated site were obtained at the time of surgery, and at 6 weeks, 3 months, 6 months and one year after the procedure. The patients were then followed for up to 4 years.

In order to assess the incorporation of the bone graft, each radiograph was reviewed by two independent examiners. The following parameters were evaluated: 1) Presence or absence of trabeculae within the grafted defect, 2) Overall bone density, 3) Quality of bone at the border of graft (described as well defined, hazy or invisible), and 4) Bone density within the defect (described as same as, equal to, or less than adjacent normal bone).

Incorporation was said to be present if the border between graft and cavity was vague and trabeculae were present at the interface. Consolidation was identified if the border between graft and cavity was not identifiable, trabeculae were present at the interface, and the density of bone graft was the same as that of the adjacent normal bone. Failure was determined to have occurred when a secondary operation to revise the primary bone graft procedure was needed or when there was no radiographic evidence of graft incorporation. The follow-up interval was defined as the period of time from the last operative intervention to the last clinical and radiographic evaluation.

In addition to the radiological data, variables analyzed included: patient age, gender, diagnosis, site of disease, defect size, procedure performed, presence or absence of a preoperative fracture, and complications related to surgery. A notation was made about the type of defect, either open or closed. A closed defect was defined as a defect within bone with well contained graft whereas an open defect was generally located on the surface of bone and bordered only by periosteum or muscle.

Data was stored and collated on Microsoft Excel software and then transferred to SPSS software for analysis.

## RESULTS

Diagnostic categories included: cystic (25), fibrous (15), chondroid (36), giant cell lesions (14), osteomyelitis (1), osteonecrosis (1), osseous tumor (3), lymphoma (1) and cavitary non-union (1) There were 65 benign latent lesions, 27 benign aggressive tumors and 5 low-grade malignant tumors. The most common sites of disease were distal femur, proximal tibia, proximal humerus, pelvis and distal tibia (Table **1**).

The mean defect size was 42.8 cm^3^ (0.48 - 315.0 cm^3^). There were 87 (89.7%) closed defects and 10 (10.3%) open defects. In all cases the tumor was curetted; in 44.5% a high speed burr was used, and in 45.5% phenol was applied to extend the margin of resection. The defect was filled with particulate cortical allograft in all cases. Instrumentation was used to stabilize the bone in 11% of cases. Thirteen (13.4%) of 97 patients had a pathologic fracture prior to treatment.

The rate of consolidation was 60%, from 6 weeks to 108 weeks, and the rate of incorporation was 91%, from 4 to 68 weeks. There was no statistically significant difference between osseous healing and diagnosis, defect size or site of disease. An example of a patient with a bone cavity filled with microparticulate graft is given in Fig. (**2**).

Eleven procedures, in eleven patients, were considered failures. There were seven patients with recurrent tumors; three with giant cell tumors, two with aneurysmal bone cysts, and one each with a fibroxanthoma and fibrous dysplasia (Table **2**).

The average time from the index procedure to tumor recurrence was 9.7 months. All patients, but one underwent a secondary grafting procedure with satisfactory functional and oncologic outcomes. One patient with a giant cell tumor of the distal radius, in addition to having a recurrence, sustained a closed fracture after the index procedure which healed with cast immobilization. In addition, this patient had a soft tissue recurrence following re-excision and grafting. She is free of tumor three years after the bone recurrence and two years six months after the soft tissue recurrence (Fig. **3**). The defect is consolidated.

Another patient with a giant cell tumor of the distal tibia presented with a recurrence after an incomplete resection at an outside hospital. At the time of treatment for this recurrence, there was a 1 cm defect in the articular cartilage that was filled with a gel foam patch and packed with microparticulate bone allograft. The tumor recurred again after 14 months. At that time, he underwent re-curetting with bone allograft and polymethylmethacrylate packing of the defect. He is free of tumor twenty months following treatment for the second recurrence. Finally, a 44 year old patient with fibrous dysplasia of the proximal femur and with long-standing deformity of the proximal femur complained of persistent pain and inability to walk without assistive devices following curetting and allograft packing. This patient underwent resection of the proximal femur and reconstruction with an un-cemented proximal femoral replacement.

Two patients had deep infections that required operative debridement and antibiotic therapy. In both patients, the infection resolved and the graft was retained and subsequently consolidated. One patient had a cystic chondroblastoma of the calcaneus, and the other had a chondrosarcoma of the pelvis that was resected and instrumented with segmental pedicle screws and plates. In the later case, the allograft was used to fill a large defect between the residual sacrum and the ilium.

Two additional patients had prolonged drainage and required oral antibiotics. A third had a stitch abscess that resolved with oral antibiotics and local wound care.

Two patients required re-operations, for a non-union of the femur in one case, and an acetabular fracture in another. The patient with the non-union presented with a large unicameral bone cyst in the inter-trochanteric region of the hip with extension into the sub-trochanteric femur. In addition, he had a displaced pathologic fracture. This patient underwent curetting and allograft bone packing and stabilization with a hip screw and side plate. Due to persistent pain and the radiographic presence of a failure to heal six months post-operatively, this patient underwent removal of the screw and side plate and antegrade intramedually nailing The unicameral cyst was completely healed and the fracture united 8 weeks following the revision procedure. In addition, a 77 year old male, with a protrusio fracture of the pelvis, following chemotherapy and radiation for a non-Hodgkin’s lymphoma of the hip, underwent total hip arthroplasty and augmentation of the medial acetabular deficiency with microparticulate allograft. He subsequently developed a large medial wall acetabular fracture with a protrusio deformity for which he underwent acetabular cage reconstruction with a large structural allograft.

Finally, a 19 year old male with osteonecrosis of the proximal tibia developed regional pain syndrome after curetting and allograft bone grafting of the defect. This resolved two years after the index procedure with non-operative pain management.

## DISCUSSION

Clinical results obtained in this study indicate that freeze-dried particulate allografts can be safely used for filling of cavitary bone defects.

In this study, all allografts were freeze-dried and, during processing, the machine temperature was kept cooler than 50°C. The freeze-drying process, removal of fat and marrow elements and standardization of the milling to near exact particle size, resulted in a stronger compacted graft substrate that is more resistant to shear, the usual mode of mechanical failure [24, 25]. These reproducible osteoconductive and osteoinductive properties of the graft produce the uniform clinical results reported in this investigation. Osteoinductive and osteoconductive properties of these grafts were established in laboratory studies [1]

Both benign and malignant tumors of bone present diagnostic and therapeutic challenges for the orthopaedic oncologist. The indications for surgical treatment of benign bone tumors and tumor-like lesions depends on the presence or absence of clinical symptoms, the anatomic location of the lesion, and its biological activity. Symptomatic lesions that progressively increase in size and demonstrate radiographic signs of aggressiveness such as deep endosteal scalloping, cortical destruction and periosteal new bone formation, should be considered for biopsy and surgical removal [8]. Despite advanced imaging techniques, including magnetic resonance imaging, fastidious attention to tumor treatment principles, and the availability of allogenic grafts and bone substitutes, control of tumor recurrence and osseous healing can be problematic.

Despite extended curetting and the application of phenol for patients with benign aggressive tumors, there were seven tumor recurrences in this series. In six of these patients, salvage was achieved by appropriate surveillance and early intervention by repeated intralesional excision and allograft bone grafting. In the 11 patients with giant cell tumors, there were 3 recurrences (27%). O’Donnell [12] reported fifteen recurrent tumors in 60 patients (25%), and Malawer recorded eight recurrent tumors in 103 (7.9%) patients [26]. Although thorough curetting, cryosurgery and the use of a high speed burr extended the intralesional margin of resection these newer techniques have not eliminated the incidence of tumor recurrence. This highlights the fact that neither bone grafting nor the improvement in surgical techniques have solved this problem.

We considered the seven cases with tumor recurrence as failures. However, this is not altogether accurate since in six of these patients, treated with re-resection and bone grafting, bone incorporation was complete. Even two patients with deep infections retained and incorporated their grafts. In two cases results with allograft bone were not adequate. The first patient was an elderly man with a lymphoma who underwent chemotherapy and radiotherapy to the acetabulum. He subsequently developed avascular necrosis and a protrusio deformity of the proximal femur. Despite the appearance of graft consolidation after total hip arthroplasty, the patient subsequently developed a fracture of the medial wall requiring revision surgery with a structural allograft and a cage. Although a number of successful grafting procedures using allograft particulate bone have been performed in patients with deficient medial acetabular walls, radiotherapy in this patient more than likely inhibited bone remodeling. Although the patient with fibrous dysplasia underwent internal fixation to augment the proximal femur after curetting and allograft packing, he continued to have pain that may have been more related to his disease than to structural insufficiency. When the oncologic results are separated from the reconstructive results, the later two cases and the two deep infections represent the only true failures of the use of the microparticulate bone allograft in the 97 patients included in this series.

In this study, the rate of consolidation was 60% (from 6 to 108 weeks), and incorporation was observed in 91% (from 4 to 68 weeks). In most cases, the short healing period made prolonged cast immobilization unnecessary. Internal fixation was used in only 11% of cases. The microparticulate graft was effective for both closed and open defects and residual graft in the adjacent soft tissue resorbed between 6 weeks and 3 months. The absence of donor morbidity and the rapid rate of incorporation, coupled with a low complication rate, make the microparticulate bone allograft an attractive alternative to autograft. The bone allograft composite used in the study facilitated rapid vascular in-growth. This rapid process of neo-vascularization and incorporation may explain the relative resistance of this graft material to peri-operative bacterial contamination and preservation of the graft in the face of deep infection.

The present study was performed with a limited objective. We wanted to find out if replacing autologous bone grafting in patients with cavitary defects following resection of tumors was a sound clinical procedure. It appears it is. Results with autografts are well known. Success rate as also high as it is with a present technique. With success rates as high as these comparisons have little to offer. The literature on the treatment of bone defects created by tumor ablation is scant. Comparison of results obtained in the present study with those obtained with revision arthroplasty would fall short of the mark, as the procedures and the reasons for performing operation are vastly different.

## Figures and Tables

**Fig. (2):**
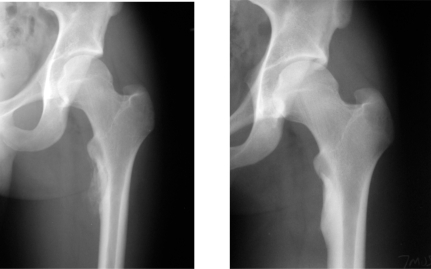
AP radiograph of femur of 23 year old male with an aneurysmal bone cyst involving the subtrochanteric region of the femur following resection and allograft packing. **Left:** incorporation at 6 weeks. ** Right:** consolidation at 3 months.

**Fig. (3A):**
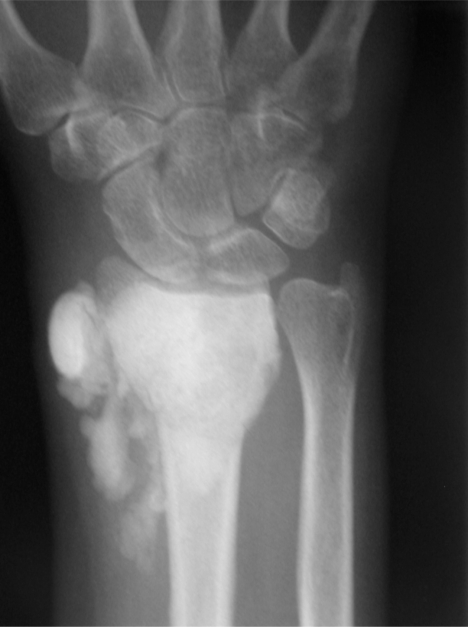
Post-operative AP radiograph of the wrist of a 51 year old woman with a giant cell tumor of the distal radius who underwent extended curetting and allograft packing. There is extensive bone graft material in the soft tissue.

**Fig. (3B):**
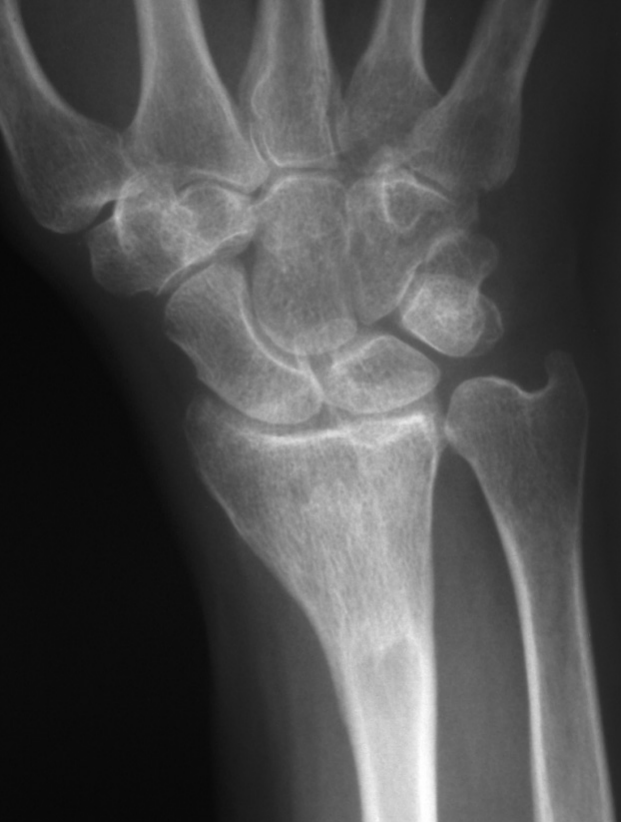
AP radiograph of the same patient one year later with complete incorporation of the graft material and resorption of the graft in soft tissue.

**Table 1:** Anatomic Locations of Lesions

Site	Number & Percent
Proximal femur	5 (5.2)
Distal femur	33 (34%)
Proximal tibia	18(18.6)
Distal tibia	6 (6.2)
Proximal humerus	8 (8.4)
Distal humerus	3 (3.1)
Proximal radius	1 (1)
Distal radius	2 (2)
Proximal ulna	1 (1)
Distal ulna	2 (2)
Phalanx	4 (4)
Cuboid	1 (1)
Calcaneus	1 (1)
Pelvis	6 (6.2)
Distal fibula	3 (3.1)
Cuneiform	1 (1)
Patella	1 (1)
Clavicle	1 (1)

**Table 2:** Patients with Tumor Recurrences

Case	Age	Tumor	Location	Previous Surgery
1	31	Giant cell tumor	Distal humerus	No
2	51	Giant cell tumor	Distal radius	No
3	62	Giant cell tumor	Distal tibia	Yes
4	18	Aneurysmal bone cyst	Proximal radius	No
5	14	Aneurysmal bone cyst	Distal tibia	No
6	12	Fibroxanthoma	Distal tibia	No
7	44	Fibrous dysplasia	Proximal femur	Yes
